# New insights into contrast-associated acute kidney injury: the key role of endothelial dysfunction

**DOI:** 10.3389/fneph.2025.1582775

**Published:** 2026-01-20

**Authors:** Mohamed Fakhfakh, Taha Lassoued, Firas Nouri, Nizar Ibn El Mechri, Ala Daly, Salem Abdessalem, Souad Ferjani, Sami Milouchi

**Affiliations:** 1 Cardiology Department, Habib Bourguiba Hospital Medenine, Medenine, Tunisia; 2 Clinic Pasteur Tunis, Tunis, Tunisia; 3 Radiology Department, Habib Bourguiba Hospital Medenine, Medenine, Tunisia

**Keywords:** contrast-associated acute kidney injury, endothelial dysfunction, microcirculation, percutaneous coronary interventions, prediction

## Abstract

**Background:**

Contrast-Associated Acute Kidney Injury (CA-AKI) is a major cause of acute kidney injury in hospitalized patients, which is triggered by the administration of iodinated contrast agents during computed tomography scans and angiographic procedures. It significantly elevates cardiovascular risk and stands as a major complication of coronary angiography, contributing to a marked deterioration in patient prognosis with elevated rates of morbidity and mortality.

**Aim:**

Our main goal was to assess the predictive factors of CA-AKI and investigate a possible association between pre-existing endothelial dysfunction and the occurrence of CA-AKI following Percutaneous Coronary Interventions (PCI). We also intended to explore possible preventive measures of CA-AKI.

**Methods:**

We conducted a prospective observational longitudinal study enrolling patients with an indication for PCI. Patients underwent an assessment of renal function (baseline creatinine, 24h and 48-72h after administration of contrast agent). We also evaluated renal function at one month as a secondary endpoint. Then, we analyzed Endothelial Quality Index (EQI) by Finger Thermal Monitoring (FTM) with E4 diagnosis Polymath.

**Results:**

We enrolled 187 patients (134 males, 53 females) in our study with a mean age of 61.1± 11.8 years. Over half (56.7%) were type 2 diabetes. A total of 60 cases of CA-AKI were reported (33.7%). The mean EQI was 0.86 ± 0.61. The vast majority of our study population (n=178; 95.2%) had endothelial dysfunction (EQI<2), and a significant proportion (n=142; 75.9%) had severe endothelial dysfunction (EQI<1). In our study, CA-AKI incidence was significantly associated with severe endothelial dysfunction (p=0.007). It was also strongly correlated to the rescue PCI (p=0.002), contrast media volume>100ml (p=0.015) and the presence of a two-vessel coronary artery disease (p=0.008). In multivariate analysis, severe endothelial dysfunction (OR = 5.46; p = 0.014), rescue PCI (OR = 5.77; p = 0.04) and contrast medica volume equal or over 140 ml (OR = 6.96; p = 0.036) were independent risk factors of CA-AKI. We found that pre- and post-hydration with isotonic saline solution and that patients whose baseline treatment includes statins, were significantly prevented from developing CA-AKI. (p=0.007 and 0.008 respectively).

**Conclusion:**

Our study showed a significant association between the presence of severe endothelial dysfunction, assessed non-invasively by FTM, and the risk of developing CA-AKI. These results appear to be relevant considering that EQI is a low-cost, non-invasive and easily reproducible marker of endothelial dysfunction.

## Introduction

Contrast-Associated Acute Kidney Injury (CA-AKI) is defined as an absolute (≥0.5 mg/dl) or relative increase (≥25%) in serum creatinine after 48–72 hours from exposure to an iodinate contrast agent (1). The incidence differs from 1–2% (2–4) in patients with no risk factors, to 50% in some specific and vulnerable subgroups, such as patients undergoing percutaneous coronary interventions (PCI) or other complex procedures requiring high volumes of iodinated contrast agents (5, 6). However, the average CA-AKI incidence has decreased from 15 to 7% in the last ten years (2). This is likely attributable to an improving awareness of risk factors, taking preventive strategies and early detection. The occurrence of CA-AKI is influenced by both patient-specific risk factors and procedural details, such as high volumes of contrast media and the intra-arterial administration route.

CA-AKI typically causes mild and transient kidney impairment, and some patients may need to undergo hemodialysis, thus increasing mortality rates (7, 8). Key risk factors for CA-AKI include pre-existing renal impairment, chronic kidney disease (CKD), diabetes mellitus, congestive heart failure, myocardial infarction, advanced age, anemia and hypertension (9, 10). In renal tissue, iodinated contrast agents appear to cause transient constriction of the afferent and efferent arterioles as well as the vasa recta. In fact, arteriolar constriction usually occurs for a few seconds to minutes in the peripheral circulation (11–13), whereas this constriction can be prolonged and endure for hours in kidneys particularly in case of significant endothelial dysfunction (14, 15). This hypoxic condition may lead to a damage in renal tubules (11–13). Another potential pathogenic mechanism might involve direct toxicity to tubular cells, resulting in cellular apoptosis (15). Given the crucial involvement of renal vasoconstriction and endothelial dysfunction in the pathophysiology of CA-AKI, we considered it essential to evaluate pre-existing endothelial impairment, which may lead to this complication. In addition, several recent studies have reinforced the relevance of this pathophysiological pathway. Perrotta et al. (16) demonstrated that vascular and biochemical markers of endothelial dysfunction are strongly associated with the development of CA-AKI. Furthermore, two studies published in the European Heart Journal in 2024 (17, 18) highlighted the protective effect of inorganic nitrate supplementation, known to enhance nitric oxide bioavailability and mitigate renal oxidative stress, thereby supporting the concept that targeting endothelial dysfunction can effectively reduce the risk of CA-AKI. These findings collectively strengthen the scientific basis for investigating endothelial status as a determinant of contrast-associated renal injury in our study. This evaluation was operationalized in our research, through a non-invasive assessment of endothelial function using finger thermal monitoring (FTM) with the E4-Diagnose device (Polymath Company, Tunisia) (19). This technique provides a dynamic measure of microvascular reactivity and thermoregulatory response, allowing a practical quantification of endothelial impairment in a real-world interventional cardiology setting. Our methodological approach was selected to ensure the feasibility and the strong clinical applicability. We used a non-invasive, low-cost, and easily implementable technique, whereas previous studies mainly relied on invasive or high-cost biomarker-based evaluations of endothelial dysfunction. This protocol also facilitated the control of potential biases through standardized inclusion criteria, uniform measurement procedures and multivariable adjustment for relevant confounders, thereby strengthening the reliability and internal validity of our findings.

### Aim of the study

Our objectives were to investigate the predictor factors of CA-AKI in the context of interventional cardiology, to assess the role of the endothelial dysfunction in the development of CA-AKI and to identify the possible approaches to preventing CA-AKI.

## Materials and methods

### Study design

We carried out a longitudinal, prospective, descriptive, and analytical study about the patients hospitalized in the cardiology department at Habib Bourguiba University Hospital in Medenine, Tunisia. This study was realized during a 15-month period between September 2022 to December 2023.

### Patients

We enrolled in our study consecutive patients of both sexes, aged 18 or older, hospitalized for a coronary procedure (coronary angiography or angioplasty), and who signed a written formal consent.

Exclusion criteria included patients under 18 years old, patients with end-stage chronic kidney disease (creatinine clearance <15 mL/min/1.73 m²) or on hemodialysis, severe chronic heart failure (left ventricular ejection fraction <20%), arteriovenous fistula, upper limb amputation, history of Raynaud’s syndrome, and patients who developed acute heart failure upon admission or during hospitalization requiring diuretic therapy. In fact, patients with Raynaud’s phenomenon were excluded, as the pathological vasospasms characteristic of this condition can profoundly alter digital blood flow and temperature responses, rendering finger thermal monitoring results unreliable and potentially unsafe.

Additionally, patients were excluded if they had contrast exposure within the previous seven days, experienced cardiogenic shock during hospitalization, had an invalid endothelial function assessment, or were hemodynamically or respiratory unstable, including those who had undergone fibrinolytic therapy with documented failure. It should be noted that rescue angioplasty was defined as a procedure performed in ST-Elevation Myocardial Infarction (STEMI) patients with fibrinolytic failure confirmed clinically, electrocardiographically, and by coronary angiography.

As part of the CA-AKI prevention protocol, nephrotoxic drugs such as anti-aldosterone agents and (nonsteroidal anti-inflammatory drugs) NSAIDs were discontinued 24 hours before the procedure. Angiotensin-converting enzyme (ACE) inhibitors, angiotensin II receptor blockers (ARBs), and metformin were also withheld 24 hours prior in patients with moderate renal impairment (eGFR <60 mL/min/1.73 m²). In accordance with the European Society of Cardiology guidelines for myocardial revascularization, all patients with moderate or severe chronic kidney disease (National Kidney Foundation stages 3b and 4) and an expected contrast volume exceeding 100 mL, received intravenous hydration with isotonic saline at 1 mL/kg/h (0.5 mL/kg/h if LVEF <35% or NYHA >2) for 12 hours before and 24 hours after the procedure. A statin loading dose (80 mg atorvastatin or 20 mg rosuvastatin) was administered to all patients, including both statin-naïve and pretreated individuals (20). Medication administration and hydration were performed by nursing staff.

Clinical examination and laboratory assessments were conducted prior to the angiographic procedure.

### Test methods

#### Evaluation of microvascular circulation and endothelial function

In this study, we used the E4-Diagnose device (Polymath Company, Tunisia), employing a fully automated and standardized post-occlusion reactive hyperemia (PORH) procedure based on a protocol similar to that of the TUN-EndCOV study (21). The E4-Diagnose is a non-invasive tool for measuring skin temperature with exceptional precision (0.002°C), featuring a portable microcontroller (MCU), two high-accuracy finger temperature sensors, and an integrated wrist cuff. The automated processes and calculations were handled by the MCU’s embedded firmware, with data being visualized, stored, and exported via specialized PC software.

Testing was performed in a controlled setting, with the room dimmed and quiet and ambient temperature maintained between 22–24°C. Participants were instructed to fast, avoid smoking, and refrain from heavy physical activity for at least four hours before the test. They were given at least 20 minutes to acclimatize, remaining seated and relaxed. Systolic blood pressure was kept below 160 mmHg, and the index finger temperature was confirmed to be above 27°C. The wrist cuff was positioned on the dominant forearm, and the finger sensors were firmly attached to both index fingers.

In alignment with the TUN-EndCOV study (21), the Endothelial Quality Index (EQI) was determined to be the optimal marker for endothelial function, with classification thresholds as follows:

EQI ≥ 2: Indicative of good endothelial function.
EQI < 2: Suggestive of endothelial dysfunction.

A threshold of two for endothelial dysfunction was consistent with previous studies (22) and was statistically validated. Further stratification of the endothelial dysfunction group was done using a cut-off value of one for a more detailed analysis.

Mehran risk score was calculated based on clinical and biological findings to predict the risk of CA-AKI post PCI (8).

#### Laboratory findings

In this study, we used the CKD-EPI equation without race to calculate the Estimated Glomerular Filtration Rate (eGFR). We applied the definition of CA-AKI most commonly used in the literature, which corresponds to an increase in serum creatinine greater than 44 µmol/L and/or more than 25% from the baseline within 48 to 72 hours after iodinated contrast exposure. Recently, the Francophone Society of Nephrology, Dialysis and Transplantation, aligned with KDIGO 2012, defines CA-AKI as an increase in serum creatinine of at least 0.3 mg/dL, equivalent to 26.5 µmol/L, or an increase of at least 1.5 times the baseline value within the same time frame. This definition was not used in our analysis (23). Serum creatinine was measured at 24 hours, 48 hours and again in one month after coronary angiography. The latter being considered a secondary endpoint.

Chronic kidney disease staging was based on estimated glomerular filtration rate. Stage 3 indicated moderate to moderately severe renal impairment and was subdivided into stage 3a (eGFR between 45 and 59 mL/min per 1.73 m²), and stage 3b (eGFR between 30 and 44 mL/min per 1.73 m²). Stage 4 corresponded to severe impairment (eGFR between 15 and 29 mL/min per 1.73 m²), and stage 5 represented a kidney failure (eGFR below 15 mL/min per 1.73 m²).

Other laboratory tests at T0 were: complete blood count (CBC), serum sodium (mEq/L), serum potassium (mEq/L), lipid profile (mmol/l) (total cholesterol, triglycerides, high density lipoprotein (HDL) cholesterol), diabetes screening tests (fasting blood glucose and/or HbA1c). Other markers were also screened: (creatine phosphokinase CPK(U/L), lactate dehydrogenase LDH (U/L), serum uric acid (mg/dl)). These parameters were measured in all patients.

#### Echocardiography

A complete echocardiographic evaluation of the systolic and diastolic left ventricular (LV) function was performed using a General Electric (GE) Vivid S6 ultrasound machine with a 5 MHz cardiac probe.

#### Statistical analysis

Continuous variables were presented as mean ± standard deviation (SD), while categorical variables were reported as absolute frequencies and percentages. Group differences were assessed using either the Student’s t-test or the Mann–Whitney U test for continuous variables. For categorical variables, differences were analyzed using chi-square test and Fisher’s exact test. Differences in percentages were evaluated using the chi-square test, while differences in means were assessed with the Student’s t-test. Risk ratios were estimated with 95% confidence intervals (CI). Variables that were statistically significant in univariate analysis, as well as those deemed clinically relevant with a 10% risk of error, were included in a multivariable logistic regression model to identify independent predictors of CA-AKI, and to determine adjusted odds ratios (OR). The receiver operating characteristic (ROC) analysis was performed to establish the cut-off values for continuous variables associated with endothelial dysfunction. A p-value of <0.05 was considered statistically significant. Statistical analysis was performed using IBM SPSS (Statistical Package for Social Science) Statistics version 22. Missing data were handled using complete-case analysis, whereby only participants with available data for the relevant variables were included in each analysis, ensuring the robustness and validity of the results.

## Results

A total of 187 consecutive patients (134 males, 53 females), with a mean age of 61.1 ± 11.8 years, were enrolled on the study. Our population was at high cardiovascular risk, 60.4% of patients were smokers, 49.2% had arterial hypertension, 56.7% had diabetes, 37.2% had dyslipidemia, and 13.9% had a history of coronary disease.

The mean baseline eGFR was 73.67 (± 20.08) ml/min. Moderate renal impairment (CKD stage 3), was present in 24.2% of patients. No patients had severe chronic kidney disease (CKD stage 4). The incidence of CA-AKI in our population, based on the classic definition (within 48–72 hours), was 33.7% (n=60). Renal function deterioration at 24 hours occurred in 32.8% of patients (n=19), with only three of them demonstrating a return to normal renal function at 48 hours post-procedures. Delayed AKI assessed after one month was observed in 21.2% of patients, with two-thirds (n=20) experiencing persistent renal impairment after initial worsening within 24 to 48 hours post-procedure. The remaining nine patients exhibited late deterioration in creatinine levels.

The Mehran risk score was on average 5.13 ± 3.41 and 13% had a score exceeding 10. ST segment elevation myocardial infarction (STEMI) was the principal indication for performing coronary angiography in our population with 46.5% of cases. As for the contrast agents, Iopromide (Ultravist*) was administered to 138 patients (74.2%), Iodixanol (Visipaque*) to 35 patients (18.3%), and Iohexol (Omnipaque*) to 13 patients (6.5%). The analysis of endothelial function revealed an EQI index averaging 0.81 ± 0.64. Nearly the entire study population (n=178; 95.2%) had endothelial dysfunction (EQI <2), with around two-thirds (n=142; 75.9%) experiencing severe endothelial dysfunction (EQI <1). The demographics, clinical characteristics, endothelial function, and echocardiographic findings of the study population at the baseline, categorized by the development or non-development of CA-AKI within 48–72 hours, are presented in **Table 1**.

**Table 1:** Patients’ demography and clinical characteristics.

Characteristics	CA-AKI+ (n=60)	CA-AKI- (n=118)	p-value
Age >63 years, n (%)	27 (45)	58 (49.2)	0.63
Male sex, n (%)	41 (68.3)	86 (72.9)	0.59
Obesity, n (%)	29 (48.4)	39 (33)	0.054
Hypertension, n (%)	28 (46.7)	58 (49.2)	0.874
Diabetes, n (%)	30 (50)	71 (60.2)	0.205
Smoking, n (%)	18 (29.7)	43 (36.5)	0.63
Dyslipidemia, n (%)	44 (73.4)	95 (80.5)	0.34
History of coronary disease, n (%)	27 (45)	53 (44.9)	1
Systolic blood pressure (mmHg) [mean± SD]	134.1 ± 9.8	131.5 ± 13.4	0.40
Diastolic blood pressure (mmHg) [mean± SD]	74.3 ± 9.2	73.9 ± 11.6	0.86
LVEF<50%, n (%)	38 (64.1)	73 (61.7)	0.87
Coronarography indication			
STEMI/Primary angioplasty, n (%)	16 (26.7)	41 (34.5)	0.62
STEMI/Rescue PCI, n (%)	18 (30)	6 (5.5)	**0.03**
NSTEMI, n (%)	27 (45.3)	43 (36.5)	0.26
CCS, n (%)	3 (4.7)	7 (6.1)	1
Performed procedure			
Coronary angiography, n (%)	14 (23.4)	35 (29.6)	0.48
PCI, n (%)	45 (75)	80 (67.8)	0.39
Contrast Media			
CM volume >100(mL), n (%)	60 (100)	108 (91.3)	**0.015**
CM volume/ CrCl ratio>3.7, n (%)	3 (5)	14 (12)	0.18
Iohexol (Omnipaque*), n (%)	4 (6.3)	7 (6.2)	1
Iopromide (Ultravist*), n (%)	48 (79.4)	86 (72.6)	0.36
Iodixanol (Visipaque*), n (%)	9 (14.3)	25 (21.2)	0.316
Mehran Score≥ 6, n (%)	33 (54.7)	57 (48.2)	0.43
Medications			
ACE inhibitors/ARBs, n (%)	47 (78.1)	88 (74.8)	0.71
Diuretics, n (%)	5 (7.8)	14 (12.2)	0.45
Anti-aldosterone, n (%)	3 (4.7)	8 (7)	0.74
Beta-blockers, n (%)	59 (98.4)	109 (92.2)	0.09
SGLT2 inhibitors, n (%)	11 (18.8)	26 (21.7)	0.88
Antithrombotic therapy			
Aspirin, n (%)	59 (98.4)	116 (98.2)	1
Clopidogrel, n (%)	50 (82.8)	78 (66.1)	**0.023**
Ticagrelor, n (%)	10 (17.2)	29 (24.6)	0.34
Acenoucoumarol, n (%)	0 (0)	4 (3.5)	0.29
Coronary status			
One-vessel-disease, n (%)	14 (23.8)	32 (27)	0.72
Two-vessel-disease, n (%)	30 (49.2)	33 (27.9)	**0.008**
Three-vessel-disease, n (%)	16 (27)	52 (44.1)	0.054
Endothelial function analysis			
EQI [mean± SD]	0.78 ± 0.6	0.89 ± 0.63	0.26
Flow_ratio [mean± SD]	3.97 ± 6.97	4.72 ± 8.44	0.54
Peak_time [mean± SD]	92.71 ± 57.3	92.05 ± 68	0.94
Half_time_decay [mean± SD]	103.04 ± 104.6	102.53 ± 103.4	0.97
Endothelial dysfunction (EQI <2), n (%)	3 (4.7)	6 (5.2)	1
Severe endothelial dysfunction (EQI<1), n (%)	49 (81.3)	73 (61.7)	**0.007**
Preventive measures			
Hydration, n (%)	1 (1.7)	16 (13.6)	**0.013**
Statin loading dose, n (%)	57 (95)	111 (94.1)	1
On chronic statin therapy, n (%)	13 (21.7)	48 (40.7)	**0.012**

ACE, angiotensin-converting enzyme; ARB, Angiotensin II Receptor Blocker; CCS, Chronic Coronary Syndrome; CA-AKI, Contrast-Associated Acute Kidney Injury (+, presence, - absence); CM, Contrast Media; CrCl, Creatinine Clearance; CV, cardiovascular; EQI, endothelial quality index; LVEF, Left Ventricle Ejection Fraction; NSTEMI, Non-ST-Elevation Myocardial Infarction; PCI, Percutaneous Coronary Intervention; SGLT2, Sodium-Glucose Co-Transporter 2; STEMI, ST-Elevation Myocardial Infarction. The values ​​in bold are statistically significant.

Comparative analysis of the baseline clinical laboratory tests on the patients with (CA-AKI+) and without CA-AKI(CA-AKI*−*) are shown in **Table 2**.

**Table 2:** Baseline clinical laboratory tests in our population.

Laboratory tests	CA-AKI+(n=60)	CA-AKI-(n=118)	p-value
CRP >3(mg/dl), n (%)	34 (56.3)	70 (59.4)	0.83
Anemia, n (%)	14 (23.4)	27 (22.6)	1
WBCs≥ 10.000, n (%)	18 (30)	29 (24.6)	0.47
High total cholesterol, n (%)	2 (3.7)	9 (7.8)	0.5
High TG, n (%)	21 (35.2)	28 (23.7)	0.139
Hight LDL cholesterol, n (%)	53 (88.6)	98 (83)	0.45
CKD stage 3a (45≤ eGFR ml/min/1.73 m2 < 60), n (%)	8 (12.5)	32 (28.1)	0.024
CKD stage 3b (30≤ eGFR ml/min/1.73 m2 < 45), n (%)	4 (6.3)	9 (7.9)	0.773
Hyperuricemia, n (%)	8 (13.3)	10 (8.7)	1
uncontrolled diabetes, n (%)	29 (48.1)	48 (40.8)	0.4

CA-AKI, Contrast-Associated Acute Kidney Injury; CRP, C-Reactive Protein; LDL, Low-density cholesterol, eGFR, estimated glomerular filtration rate; MRI, moderate renal impairment; TG, Triglycerides; WBCs, White Blood Cells.

In the analytical analysis, significant predictors of contrast-associated acute kidney injury within 48–72 hours were identified, including rescue angioplasty (p=0.002).

Additional factors significantly associated with contrast-associated acute kidney injury included contrast volume >100 mL (p=0.015) and a two-vessel coronary artery disease (p=0.008) (**Table 1**). Moreover, each additional milliliter of contrast was associated with a 1% rise in CA-AKI risk (OR = 1.01, p=0.02).

Regarding patients with CKD stage 3b, CA-AKI occurred in 6.3% of cases versus 7.9% among those without the complication (p = 0.77), indicating no significant association at this stage. Furthermore, in CKD stage 3a, CA-AKI was documented in 12.5% of patients compared with 28.1% in those who did not develop CA-AKI (p = 0.024) (**Table 2**).

These findings appear to contradict the existing evidence, which consistently identifies CKD as a major risk factor for CA-AKI. This discrepancy is most likely attributable to selection bias, as patients with CKD received lower contrast volumes and benefited from stricter preventive measures, including optimized hydration protocols and systematic withdrawal of nephrotoxic agents.

Early CA-AKI risk within 24 hours was notably influenced by clopidogrel (p<0.05), while ARBs showed a trend towards increased risk (p=0.083) and ticagrelor showed potential protection (p=0.076). Long-term, at one month time, ARBs treatment exhibited reduced delayed AKI risk (p=0.07), and Iohexol was significantly linked to delayed AKI (p=0.048), whereas Iopromide indicated potential protection (p=0.06), with iodixanol showing no association. Details are summarized in **Table 3**.

**Table 3:** Key CA-AKI factors at 24h and 1 month with comparative insights across timeframes.

CA-AKAI factors	Total	CA-AKI «24h» N=58		CA-AKI «48h» N=178		Delayed AKI «1 month» N=137	
	Group	CA-AKI+ (n=19)	p	CA-AKI+ (n=60)	p	AKI+ (n=29)	p
ARBs (%)	Yes No	66.7 28.8	0.083	27.3 34.1	0.753	0 23.2	0.07
Clopidogrel (%)	yes No	45.7 13	0.011	37.5 24	0.112	21.7 20	1
Ticagrelor (%)	yes No	15.8 41	0.076	30.8 34.5	0.706	17.1 22.5	0.634
Primary angiolpasty (%)	yes No	0 52.4	0.012	29.6 37.9	0.805	12.5 24.4	0.34
Contrast media (%)	-Iodixanol (Visipaque*) -Iopromide ou Iohexol	36.4 34.8	1	27.3 37.8	0.316	27.6 20.6	0.452
	-Iohexol (Omnipaque*) -Iopromide ou Iodixanol	0 36.4	0.536	36.4 35.8	1	50 19.8	0.042
	-Iopromide (Ultravist*) -Iohexol ou Iodixanol	36.4 30.8	1	37.9 29.5	0.367	17.5 33.3	0.066

CA-AKI, Contrast-Associated Acute Kidney Injury; ARBs, Angiotensin II Receptor Blockers.

In multivariate analysis, severe endothelial dysfunction (EQI <1) (OR = 5.46, 95% CI = 1.557–17.482, p = 0.014), rescue PCI (OR = 5.77, 95% CI = 1.083–30.818, p = 0.04), and contrast volume greater than or equal to 140 ml (OR = 6.96, 95% CI = 1.133–42.766, p = 0.036) were independent risk factors of contrast induced nephropathy as shown in **Table 4**.

**Table 4:** Associated factors to CA-AKI in multivariate analysis.

Variable	Odds ratio, 95 CI%	*P-value*
Rescue PCI	5.77,[1.083 -30.818]	0.040
Contrast volume ≥140 ml	6.96,[1.133- 42.766]	0.036
Severe endothelial dysfunction (EQI<1)	4.46,[1.557 -17.482]	0.014

CI, Confidence Interval; EQI, endothelial quality index; PCI, Percutaneous Coronary Intervention; ml: millilitres.

## Discussion

In our study, the incidence of CA-AKI was 33.7%, which is in accordance with published series (2, 6). CA-AKI is a major cause of hospital-acquired acute renal failure, triggered by iodinated contrast during CT scans and angiographic procedures. Recent studies (24–27) provide updated insights into its epidemiology, pathophysiology, and risk factors, highlighting the roles of endothelial dysfunction, oxidative stress, and renal hemodynamic alterations. Bartholomew et al. reported a mortality of 14% in patients with CA-AKI compared to 1.1% in those without. This represents a ten-fold rise (2).

Multiple mechanisms underlie the development of CA-AKI. Intra-arterial iodine contrast induces transient vasodilation mediated by nitric oxide release from endothelial cells (15). This is quickly followed by vasoconstriction of arterioles, which is brief in peripheral circulation (11) but can persist for hours in renal microvasculature, impacting afferent and efferent arterioles and the vasa recta (15).

Patients with diabetes and CKD are particularly at risk due to their limited nephron reserve. In these patients, contrast-induced vasoconstriction further reduces renal blood flow, compromising oxygenation of the outer medulla and triggering ischemia in proximal and distal tubules (15).

Another contributing factor is direct cytotoxicity from the contrast agent on renal tubular cells. Damage to the apical and basolateral surfaces can cause cellular degeneration, apoptosis, and the release of intracellular free iron from mitochondria. This iron promotes the formation of free radicals, intensifying oxidative stress within the renal tissue (11–13).

In light of the direct toxic effects of iodinated contrast media on vascular cells, we chose the EQI marker to assess endothelial function, taking into account that endothelial dysfunction is ubiquitous and seems to affect the entire vascular endothelium (28). This parameter is derived from the advanced ‘E4-Diagnose’ device, a groundbreaking, non-invasive tool developed by Polymath Company in Tunisia. First introduced in the 2021 TUN-ENDCOV study (21), the device offers precise, portable, and operator-independent measurements of endothelial health, both in clinical and research contexts. Using a fully automated and standardized post-occlusive reactive hyperemia (PORH) protocol, the ‘E4-Diagnose’ device enables the detection of endothelial dysfunction. In our study, we assessed endothelial function at the radial artery, following the same protocol as in the TUN-ENDCOV study. The EQI was used as the core parameter, categorizing the severity of endothelial dysfunction: EQI ≥ 2 indicates healthy endothelial function, EQI between 1 and 2 reflects moderate dysfunction, and EQI < 1 represents severe dysfunction. This approach offers a novel way to link endothelial dysfunction to the risk of developing CA-AKI, improving predictions for this complication.

In our study, severe endothelial dysfunction (EQI<1) was significantly associated and an independent predictive factor to the development of CA-AKI.

Additionally, in our cohort of patients, we examined the other factors related to the patient and/or the procedure. In the literature, diabetes and chronic kidney disease are well-established risk factors for CA-AKI in the literature (29, 30). However, our study did not find a significant association. This apparent discrepancy likely reflects selection bias, as patients with CKD received lower contrast volumes and benefited from enhanced preventive measures, including optimized hydration and systematic withdrawal of nephrotoxic agents. Besides, nephrotoxic medications are well-known contributors to the development of contrast-associated acute kidney injury, exacerbating renal vulnerability in patients undergoing contrast exposure. NSAIDs, for instance, disrupt prostaglandin synthesis, leading to afferent arteriole constriction and subsequent renal hypoperfusion, a key mechanism underlying contrast-induced nephrotoxicity (31).

In our study, certain medications, including Clopidogrel (p = 0.011) and angiotensin receptor blockers (ARBs), (p = 0.083), were associated with an increased risk of early CA-AKI onset within the first 24 hours post-contrast administration. Interestingly, while ARBs were linked to a higher early phase CA-AKI incidence, a trend toward reduced delayed AKI occurrence emerged at one-month follow-up, with no cases reported in ARB users compared to 23.2% in non-users (p = 0.07). This dual effect suggests an initial risk but a potential long-term protective role that warrants further investigation.

Furthermore, Ticagrelor showed a protective trend against early CA-AKI (p = 0.076), though this finding did not reach statistical significance. These observational results should be interpreted cautiously. Further prospective and mechanistic studies are warranted to clarify the role of these medications in the development of CA-AKI.

Moreover, the literature highlights that factors such the type and volume of contrast media, the urgency of the procedure, and the presence of coronary artery disease are associated with an increased risk of contrast-induced acute kidney injury (32–34). In our cohort, both the type and volume of contrast media emerged as significant predictors of CA-AKI. Iohexol (Omnipaque*) was significantly associated with delayed CA-AKI (p = 0.048), while Iopromide (Ultravist*) showed a trend toward protection (p = 0.06). These differences likely reflect their physicochemical properties: Iohexol’s higher osmolality and viscosity may increase renal tubular stress, reduce medullary perfusion, and enhance oxidative injury, whereas Iopromide’s lower osmolality and viscosity may better preserve renal hemodynamics. This mechanistic perspective helps to explain the observed variation in nephrotoxic potential among contrast media. Regarding contrast volume, exceeding 100 mL was strongly correlated with CA-AKI (p=0.015), while multivariate analysis confirmed that volumes surpassing 140 mL significantly increased CA-AKI risk, with an odds ratio of 6.9 (p=0.036). These findings emphasize the dual importance of selecting optimal contrast media and strictly managing contrast volume to minimize renal complications.

Beyond contrast characteristics, the coronary anatomy itself played a pivotal role in determining CA-AKI risk. Patients with 2-vessel disease (2VD) demonstrated a significantly higher risk of CA-AKI, while those with 3-vessel disease (3VD) paradoxically appeared to be protected. This unexpected relationship underscores the intricate interplay between coronary disease extent, procedural complexity, and renal outcomes. Such findings highlight the potential of CT angiography to further refine risk stratification and guide preventive strategies in clinical practice.

Adding another dimension, procedural urgency had a profound impact on CA-AKI development. Patients undergoing rescue PCI exhibited a markedly higher CA-AKI occurrence (75%) compared to those who did not (26%) (p<0.01). Multivariate analysis identified rescue PCI as an independent risk factor for CA-AKI, with an odds ratio of 5.7 (95% CI: 1.2–26). Paradoxically, primary angioplasty was associated with a protective effect against early CA-AKI (p=0.012), contrasting with existing literature suggesting higher risks during emergency interventions. This apparent contradiction may reflect our proactive monitoring and early detection strategies, which mitigated the risks typically associated with emergency procedures.

Furthermore, prevention is crucial due to the poor prognosis of CA-AKI. Song et al.’s study investigated the association between post-procedural oral hydration and the risk of CA-AKI in STEMI patients undergoing primary PCI. This study found that adequate oral hydration post-procedure was linked to a reduced risk of CA-AKI in these high-risk patients (6, 35). In our study, pre- and post-hydration significantly reduced the incidence of CA-AKI. As for statins, their role in preventing CA-AKI in patients undergoing coronary angiography remains controversial (36, 37).

In our study, a loading dose of statins had no effect on prevention, but patients who were already on chronic statin therapy developed significantly less CA-AKI.

### Strengths, limitations and perspectives

This study provides novel insights into the relationship between endothelial dysfunction and contrast-associated acute kidney injury (CA-AKI), notably highlighting non-invasive endothelial assessment as a potentially valuable predictor. The robustness of the statistical analysis further supports the reliability of our findings.

Nevertheless, several limitations should be acknowledged. The study was conducted on a relatively small observational cohort, which limits generalizability and precludes causal inference. Moreover, AKI was defined using older criteria rather than the currently accepted KDIGO definition, potentially resulting in patient misclassification. Besides, in some patients, AKI may not have been directly related to contrast exposure but rather to underlying cardiac conditions (heart failure, cholesterol atheroembolism, …) or other iatrogenic interventions, such as medications administered. Furthermore, endothelial function was assessed using the EQI, a research tool rather than a widely established clinical method, which restricts its immediate clinical applicability.

Despite these limitations, the findings suggest that endothelial dysfunction could serve as a clinically relevant risk factor for CA-AKI. Building on these results, larger multicenter studies are warranted to validate the observations and to better evaluate their impact on morbidity, mortality, and hospital stay. Furthermore, a deeper understanding of the mechanistic link between endothelial dysfunction and CA-AKI may facilitate the development of targeted preventive strategies, including the potential use of endothelial-protective therapies in high-risk patients undergoing coronary angiography, ultimately aiming to reduce the incidence and severity of CA-AKI.

Based on our findings, we propose incorporating pre-procedural assessment of endothelial function into the protocol for patients scheduled for coronary angiography to better predict CA-AKI and guide preventive measures. These include optimal peri-procedural hydration, statin loading, avoid using Iohexol and opting for an iso or low-osmolar contrast media, with limiting contrast volume to less than 100 mL.

For patients identified with endothelial dysfunction, strict control of cardiovascular risk factors—such as diabetes, hypertension, and dyslipidemia—is recommended, alongside smoking cessation and regular physical activity. Supported by the TUN END COV study (38), which demonstrated improved endothelial function after three weeks of Soludexide treatment, and recent evidence on inorganic nitrate supplementation enhancing nitric oxide bioavailability and reducing renal oxidative stress (17), we suggest considering these interventions in non-urgent cases.

Finally, we plan to implement this prevention program across multiple centers to validate its effectiveness, inform clinical guidelines, and guide public health policies, with potential extension to other imaging modalities, such as cardiac CT, increasingly recommended by the ESC.

## Conclusion

Our study demonstrated that pre-existing severe endothelial dysfunction serves as a powerful predictor of CA-AKI. It is evident that EQI analysis is an affordable, non-invasive, and highly reproducible marker for assessing endothelial dysfunction, enabling early risk identification and adapted preventive measures to enhance patient outcomes. Further research with larger patient cohorts is needed to validate these findings.

## Data Availability

The raw data supporting the conclusions of this article will be made available by the authors, without undue reservation.
