# Supplementary material for: New insights into contrast-associated acute kidney injury: the key role of endothelial dysfunction
Source: Front Nephrol. 2026 Jan 20;5:1582775. doi: 10.3389/fneph.2025.1582775 (PMC12864088; doi:10.3389/fneph.2025.1582775)
Supplement: Supplementary file 1 [file DataSheet1.pdf]

Supplementary Material- E4-Diagnose Device

The E4-Diagnose device (Polymath, Tunisia) (Figure S1) and its various parameters for assessing endothelial function have been validated against the reference device approved by the United States Food and Drug Administration (FDA), the Vendys 5000 (Endothelix, Inc., USA).

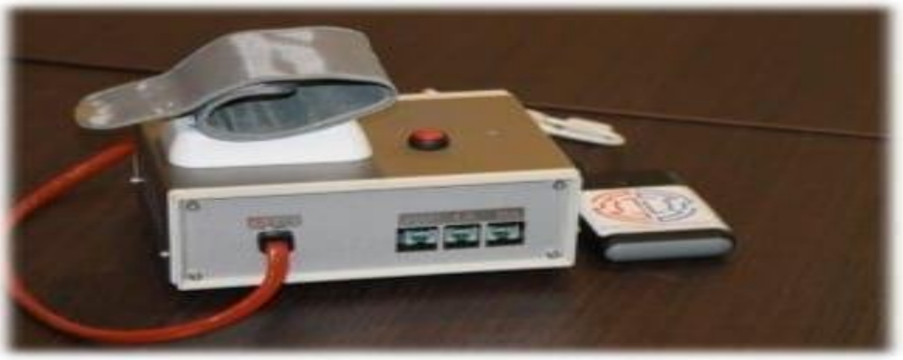

Figure S1. E4-Diagnose device.

The E4-Diagnose system is an innovative tool that enables quantitative, non-invasive, simple, rapid, low-cost, and portable assessment of endothelial function in humans. The prototype version, **E4-Diagnose Research Prototype**, offers a simplified design optimized for flexibility, ease of setup, and use, with limited but essential functionalities.

A-Device components (Figure S2):

- 1x E4-Diagnose Main Instrument Box (MIB)
- 2x Right Arm Probe (RAP)
- 2x Left Arm Probe (LAP)
- 1x Calibration/Test Probe (AMB)
- 1x External Battery (BAT)
- 1x Occlusion Cuff

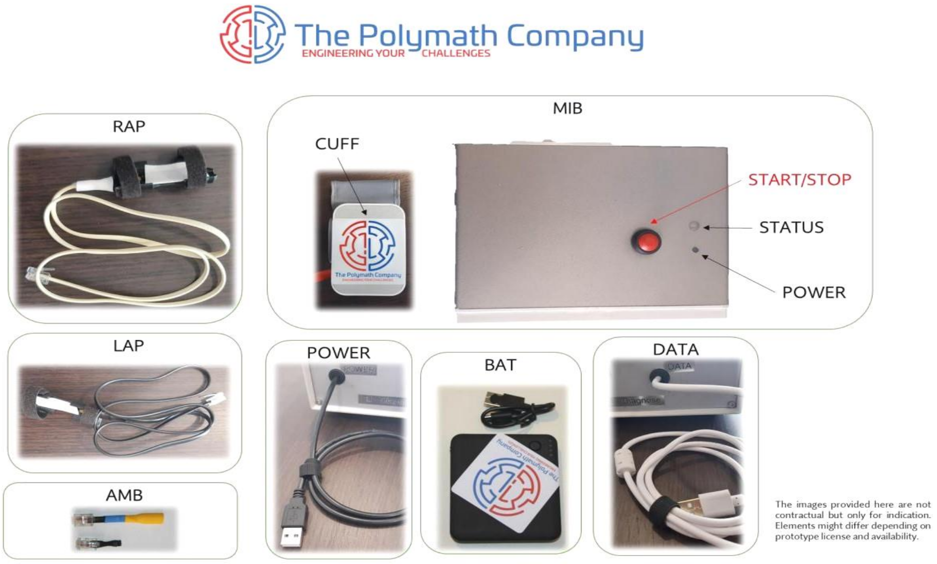

Figure S2. Components of the E4-Diagnose prototype.

B- Examination Conditions

- Maintain the ambient temperature of the examination room between 22°C and 24°C.
- Ensure patients fast for at least 4 hours; prohibit smoking and intense physical activity during this period.
- Reassure patients and warm their hands before the measurement.
- Consider measurements invalid if finger temperature is below 23°C, if temperature asymmetry between the two index fingers exceeds 3°C, or if ambient temperature varies by ≥2°C during the procedure.

C. Examination Procedure.

- Ensure the examination room is clean, with stable temperature (22–24°C) and minimal air convection.
- Seat the patient comfortably less than one meter from the prototype.
- Place the occlusion cuff on the right forearm, 1 cm above the wrist, on the same side as the palm (Figure S3).
- Remove the adhesive cover of the RAP.

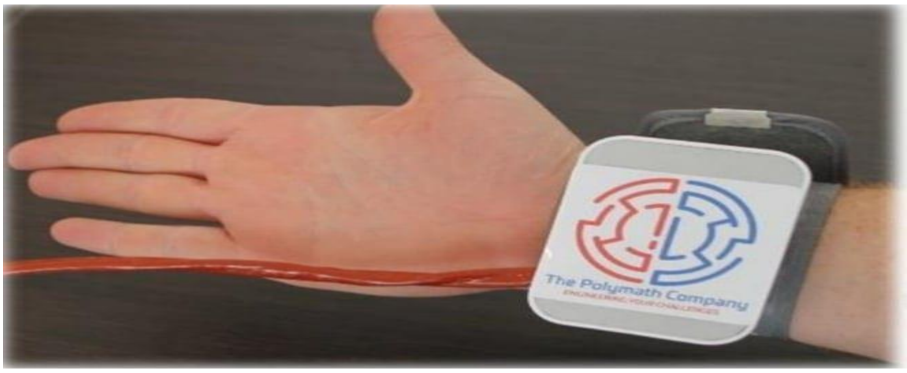

Figure S3: An occlusion cuff placed on the forearm

- Position the RAP carefully on the right index finger, aligning the digital support with the finger and centering the sensor bulb on the distal phalanx.
- Apply the adhesive firmly along the entire length of the finger to ensure optimal contact and measurement accuracy.
- Adjust the strap around the joint with moderate tension (Figure S4).
- Lightly secure the strap around the distal phalanx without applying pressure (Figure S4).

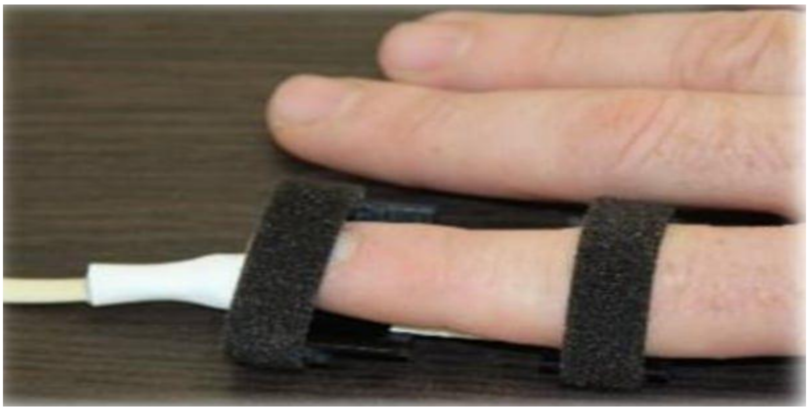

Figure S4: Sensitive thermal sensors attached to the index fingers

- Remove the adhesive cover of the LAP.
- Position the LAP carefully on the left index finger, aligning the digital support with the finger and centering the sensor bulb on the distal phalanx.
- Apply the adhesive firmly along the entire length of the finger to ensure optimal contact and measurement accuracy.
- Adjust the strap around the joint with moderate tension.
- Lightly secure the strap around the distal phalanx without applying pressure.
- Place both arms on the patient's knees, preferably with palms facing upward and wrists aligned with the knees to keep the hands free.
- Power on the battery/MIB using the Power button.
- Verify that the power indicator illuminates.
- Launch the E4-Diagnose application by double-clicking E4_Diagnosis_Launcher.
- Press the Start button on the MIB to initialize a new measurement.
- Conduct the total measurement sequence for 900 seconds **(15 minutes).**

D. Examination phases

The total measurement lasted **900 seconds (15 minutes)**, divided into three consecutive 5-minute phases:

1. Stabilization Phase

- The device detects baseline ambient and finger temperatures with the cuff deflated.
- Baseline blood flow (FLOW0) is recorded over 30 seconds.

2. Occlusion Phase

- The cuff is inflated to 50 mmHg above systolic pressure, inducing ischemia in the right hand.
- Temperature decline of the right index finger is recorded relative to the left reference finger.
- The slope of cooling is influenced by ambient temperature, skin thickness, and finger morphology.
- Compensatory microvascular vasodilation occurs physiologically during ischemia.

3. Reperfusion Phase

- The cuff is rapidly deflated, inducing immediate hyperemia due to vasodilation.
- Multiple parameters are collected during this phase.

E. Results and Parameters

- The device measures the rapid warming of the right index, with the slope proportional to blood flow.
- It calculates the difference from the occlusion phase and generates a synthetic **Endothelium Quality Index (EQI)**. Higher EQI values indicate greater NO-dependent peripheral and humeral vasodilation (Figure S5).
- EQI is dimensionless and reflects microvascular and arterial vasodilation along the arm, influenced by shear stress on the vascular endothelium.

Additional parameters recorded:

- **Peak_flow and Peak_time:** maximum blood flow and time to peak after reperfusion.
- **Half_time_decay:** time from Peak_time until blood flow decreases below half of peak flow.

Endothelial dysfunction classification (based on EQI):

- EQI > 2 → Normal endothelial function
- EQI 1–2 → Moderately impaired endothelial function
- EQI < 1 → Severely impaired endothelial function

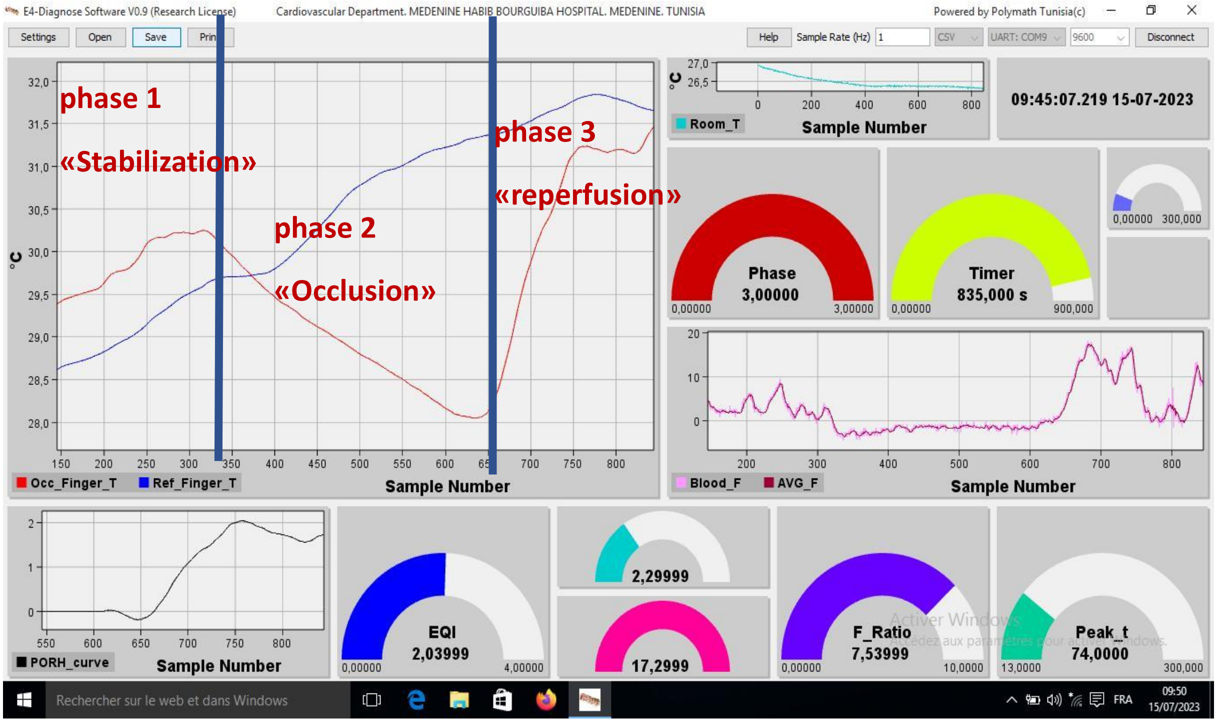

Figure S5: Final endothelial function result displayed by the E4-Diagnose device
